# Global similarity and local divergence in human and mouse gene co-expression networks

**DOI:** 10.1186/1471-2148-6-70

**Published:** 2006-09-12

**Authors:** Panayiotis Tsaparas, Leonardo Mariño-Ramírez, Olivier Bodenreider, Eugene V Koonin, I King Jordan

**Affiliations:** 1 Basic Research Unit, Helsinki Institute for Information Technology, University of Helsinki, Helsinki, Finland; 2 National Center for Biotechnology Information, National Institutes of Health, Bethesda, MD 20894, USA; 3 National Library of Medicine, National Institutes of Health, Bethesda, MD 20894, USA; 4 Present address: School of Biology, Georgia Institute of Technology, Atlanta, GA 30332, USA

## Abstract

**Background:**

A genome-wide comparative analysis of human and mouse gene expression patterns was performed in order to evaluate the evolutionary divergence of mammalian gene expression. Tissue-specific expression profiles were analyzed for 9,105 human-mouse orthologous gene pairs across 28 tissues. Expression profiles were resolved into species-specific coexpression networks, and the topological properties of the networks were compared between species.

**Results:**

At the global level, the topological properties of the human and mouse gene coexpression networks are, essentially, identical. For instance, both networks have topologies with small-world and scale-free properties as well as closely similar average node degrees, clustering coefficients, and path lengths. However, the human and mouse coexpression networks are highly divergent at the local level: only a small fraction (<10%) of coexpressed gene pair relationships are conserved between the two species. A series of controls for experimental and biological variance show that most of this divergence does not result from experimental noise. We further show that, while the expression divergence between species is genuinely rapid, expression does not evolve free from selective (functional) constraint. Indeed, the coexpression networks analyzed here are demonstrably functionally coherent as indicated by the functional similarity of coexpressed gene pairs, and this pattern is most pronounced in the conserved human-mouse intersection network. Numerous dense network clusters show evidence of dedicated functions, such as spermatogenesis and immune response, that are clearly consistent with the coherence of the expression patterns of their constituent gene members.

**Conclusion:**

The dissonance between global versus local network divergence suggests that the interspecies similarity of the global network properties is of limited biological significance, at best, and that the biologically relevant aspects of the architectures of gene coexpression are specific and particular, rather than universal. Nevertheless, there is substantial evolutionary conservation of the local network structure which is compatible with the notion that gene coexpression networks are subject to purifying selection.

## Background

The amplitude, timing, and pattern of gene expression have important phenotypic consequences, and the potential evolutionary significance of changes in the regulation and expression of genes has long been recognized [1-3]. In the last few years, high-throughput gene expression data sets from related species have accumulated, to the extent that it has become possible to study the divergence of expression in a systematic way at the genome-scale.

Initial efforts at the comparative study of gene expression divergence have yielded some interesting and unexpected results. For instance, it has been shown that the level and pattern of mammalian gene expression can evolve in a way that is both rapid and apparently unconnected to the level of functional constraint on gene sequences [4,5]. This led to the counter-intuitive suggestion that gene expression may evolve completely free of selective constraint, in other words, purely neutrally. Subsequent studies have refined the neutral view on the evolution of gene expression by demonstrating that, although selection does, in fact, constrain expression divergence, much of the observed change in expression between species may nevertheless be effectively neutral [6,7]. The potential adaptive significance of some gene expression changes has also been posited [6]. Several other recent studies have shown how patterns of gene expression, and entire regulatory networks, can quickly respond to environmental cues and substantially reorganize themselves over the course of evolution. For instance, the architecture of yeast gene regulatory networks has been shown to change dramatically in response to environmental stimuli [8], and gene expression patterns were found to diverge rapidly after gene duplication in yeast [9] and humans [10]. Prokaryotic genomes, too, show evidence of rapid, whole-sale reorganization of gene regulatory networks [11].

Given the phenotypic relevance of gene expression patterns, the apparent evolutionary lability of expression suggests that it might represent an ideal substrate on which natural selection could act to drive the functional divergence between evolutionary lineages. Indeed, comparative studies of gene expression have also uncovered intriguing connections between expression divergence and gene function. For instance, it has been shown that physically interacting proteins tend to be encoded by coexpressed genes [12,13], and that the expression levels of interacting proteins show coordinated changes across species [14]. From a broader perspective, it has been demonstrated that functionally related genes are preferentially linked in coexpression networks, and this was taken to justify the so-called 'guilt by association' heuristic whereby expression patterns are used to inform functional annotation of uncharacterized genes [15]. In a very specific example of how expression changes can lead to phenotypic divergence, the expression changes in yeast that facilitated the emergence of anaerobic metabolism have been identified and shown to be due to the evolution of a specific cis-regulatory sequence motif [16].

For the study presented here, we performed a comparative analysis of human-mouse gene expression patterns to assess the extent of expression divergence between the two species and to explore the connections between the evolution of gene expression and function. We employed the Novartis mammalian gene expression atlas [17] to compare changes in the relative expression levels between 9,105 orthologous human-mouse gene pairs across a panel of 28 shared tissues. Gene expression patterns were resolved into species-specific coexpression networks and the topological properties of these networks were compared. The interrogation of coexpression networks allows for the use of a well-developed set of analytical and conceptual tools [18-20] and provides an opportunity for the simultaneous comparison of evolution at different levels of systemic organization, i.e., global vs. local network properties. The results of this comparison indicate that human and mouse co-expression networks are indistinguishable in terms of their global properties but show drastic divergence at the local level.

## Results and Discussion

### Mammalian coexpression networks

**Figure 1:**
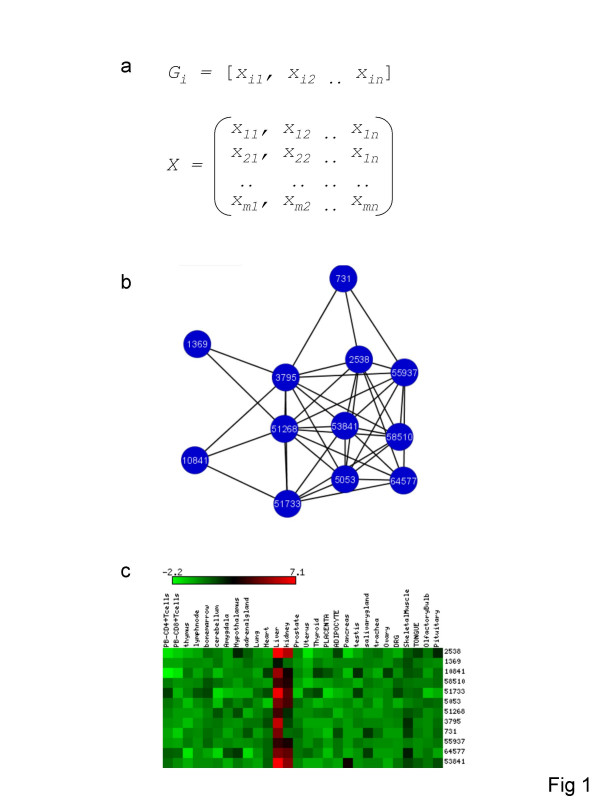
**Gene coexpression networks**. a) The expression profile of a gene *i *(*G*_*i*_) can be represented as a row vector with dimensions (*n*) equal to the number of tissues (28); all profiles taken together yield an *m *× *n *gene expression matrix (*X*) where *m *= the number of genes (9,105), *n *= the number of tissues (28) and the expression value of *Gi *in tissue *j *is represented as *x*_*ij *_[45]. b) Gene expression vectors can be compared using a number of different similarity (distance) measures such as the Pearson correlation coefficient (*r*). Genes (nodes) are connected by an edge if their vectors are sufficiently similar (e.g. *r *≥ 0.7). A relatively tightly linked cluster (subgraph) of coexpressed genes is shown. c) Visual representation of the expression patterns of the genes in this cluster underscores their similarity. Color scale based on log_2 _(*G*_*ij*_/median *G*_*i1*_...*G*_*in*_).

Tissue-specific expression profiles of human-mouse orthologous gene pairs were compared in order to evaluate the divergence of mammalian gene expression patterns. A total of 9,105 orthologous gene pairs were considered with respect to their expression levels across 28 tissues shared between the two species. All-against-all gene expression profile comparisons for the human and mouse matrices (9,105 × 28) were used to generate species-specific coexpression networks (Figure 1a). For coexpression networks, nodes correspond to genes, and edges link two genes from the same organism if their expression profiles are considered sufficiently similar (Figure 1b). A number of different metrics were used to measure the similarity (distance) between vectors of tissue-specific expression levels: Euclidean distance, Manhattan distance, Jensen-Shannon divergence, dot-product, cosine similarity and Pearson correlation coefficient. Results reported here are for networks constructed using the Pearson correlation coefficient (*PCC*). The *PCC *is widely employed for comparison of gene expression profiles and reflects similarity between expression patterns in terms of the relative expression levels across tissues. It should be noted that the results for the coexpression network analyses are qualitatively similar irrespective of the measure of profile similarity employed. Results of analyses based on the other measures of profile similarity (distance) are presented in the Supplementary Information section (see Additional file 1) along with a discussion of the relationships among those measures.

The use of the *PCC *to build coexpression networks is predicated on the choice of a threshold correlation coefficient (*r*) at, or above which, genes are considered to be coexpressed and are thus connected by an edge in the network. As previously reported [21], a series of increasing *r*-values (0.4–0.9) was evaluated for utility in building coexpression networks. When *r*-values << 0.7 are used, coexpression networks tend to congeal into graphs that are so densely connected as to preclude meaningful analysis of their topological properties. On the other hand, *r*-value thresholds ranging from 0.7–0.9 yield analytically tractable networks and qualitatively similar results. Results for coexpression networks based on an *r*-value threshold of 0.7 are reported here since this cutoff gives networks that are unlikely to contain many spurious edges but are sufficiently large and dense for robust topological analysis. For the 28-dimensional gene expression profiles evaluated here, an *r*-value of 0.7 corresponds to a highly statistically significant correlation (*P *= 3.4e-5). Furthermore, gene expression profiles with *r *≥ 0.7 can be visually appreciated to be highly similar (Figure 1c).

Human and mouse coexpression networks were evaluated with respect to a number of parameters describing their global topological properties and found to be highly similar (Table 1). The numbers of nodes and edges in each network are comparable, with the mouse network showing slighter higher values for both. The average degree (*<k*>) is the average number of edges per node and gives rough approximation of how dense the network is. The mouse network shows a slightly higher *<k*> which is consistent with the greater numbers of nodes and edges. However, *<k*> is again similar for both networks and rather high. By way of comparison, typical world-wide-web networks have <*k*>≈7. The values of *<k*> might not be particularly relevant because, as will be shown below, the degree distributions are highly skewed.

**Table 1:** Global characteristics of the coexpression networks

**Network**	**Nodes**^**1**^	**Edges**^**2**^	<***k***>^**3**^	<***C***>^**4**^	<***l***>^**5**^
Human	7,208	158,418	43.96	0.3744	4.75
Mouse	7,730	178,166	46.10	0.4003	4.80
Intersection	2,257	13,060	11.57	0.4006	6.89

^1^Number of genes (nodes) in the network – *i.e*. nodes with one or more edges

^2^Number of coexpressed gene pairs (edges) in the network

^3^Average degree (*k*), number of edges shared with other nodes, per node

^4^Average clustering coefficient (*C*) per node

^5^Average shortest path length (***l***) between any two nodes in the network

A more refined notion of network density is given by the average clustering coefficient (<*C*>). The clustering coefficient *C *of a node *i *is defined as the fraction of the pairs of neighbors of node *i *that are linked to each other: *Ci *= 2*n*_*i*_/*ki*(*ki*-1), where *n*_*i *_is the number of observed links connecting the *k*_*i *_neighbors of node *i *and *k*_*i*_(*k*_*i*_-1)/2 is the total number of possible links. The average clustering coefficient (<*C*>) is the mean of this value for all nodes with at least two neighbors, and for both the human and mouse networks <*C*>≈0.4 (Table 1). For networks of this size, these <*C*> values are considered to be quite high. By way of comparison, for randomly generated networks with the same number of edges and same degree (*k*) sequences, the expected <*C*> is estimated to be 0.0643 for human and 0.0529 for mouse. The high density of the coexpression networks is not necessarily surprising because, as one could reasonably expect, co-expression is, largely (but not entirely), transitive. In other words, if gene *A *is coexpressed with genes *B *and *C*, then genes *B *and *C *are likely to be coexpressed as well. However, the high observed values of <*C*> for the human and mouse networks do not appear to be due to the transitivity of the *PCC *similarity measure alone. This is demonstrated by the observation that networks built using the *PCC *measures between randomly permuted gene expression profiles, thus preserving some transitivity, also have values of <*C*> that are far lower than the observed values: human = 0.0933, mouse = 0.1229.

The average path length (<***l***>) is the average shortest path, or the smallest number of edges needed to connect two nodes, between any two reachable nodes in the network. Clearly, the co-expression networks exhibit "small world phenomena": on average, any two nodes are separated by only a few edges (Table 1).

**Figure 2:**
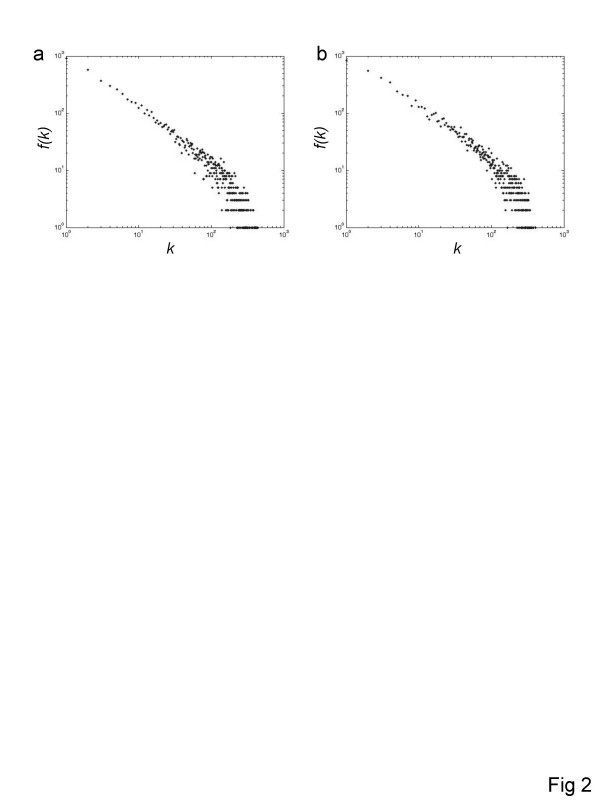
**Node degree (*k*) distributions for human and mouse gene coexpression networks**. All distributions are plotted in log_10_-log_10 _scale. Frequency distributions showing *f(k) *× *k *for human (a) and mouse (b).

Node degree (*k*) distributions were also computed for the human and mouse coexpression networks (Figure 2a and 2b). In both cases, the distribution seems to follow a power-law, that is, the probability that a randomly chosen node has degree *k*, is Pr [*K *= *k*] ∝ *k*^-α ^where the parameter *α*, is the exponent of the power law distribution. While the degree distributions seem to be well approximated by a straight line in log-log scale (α = 1.13 for the human network and α = 1.11 for the mouse network by the least squares method), there appears to be an exponential drop-off in the tail of the distributions. Thus, the distributions are more appropriately described as a fat-tailed, power-law-like distributions rather than strict power-laws. Accordingly, evolutionary models that lead to pure power-laws, typically, with α >2, such as preferential attachment, would not apply to the evolution of this network. Additional details on these distributions are provided in the Supplementary Information (see Additional file 1). Node degree distributions obtained using different distance (similarity) measures show similar fat-tailed properties and appear to be better approximated by power-laws than those obtained using the *PCC *(see Additional file 1; Supplementary Figure 3).

**Figure 3:**
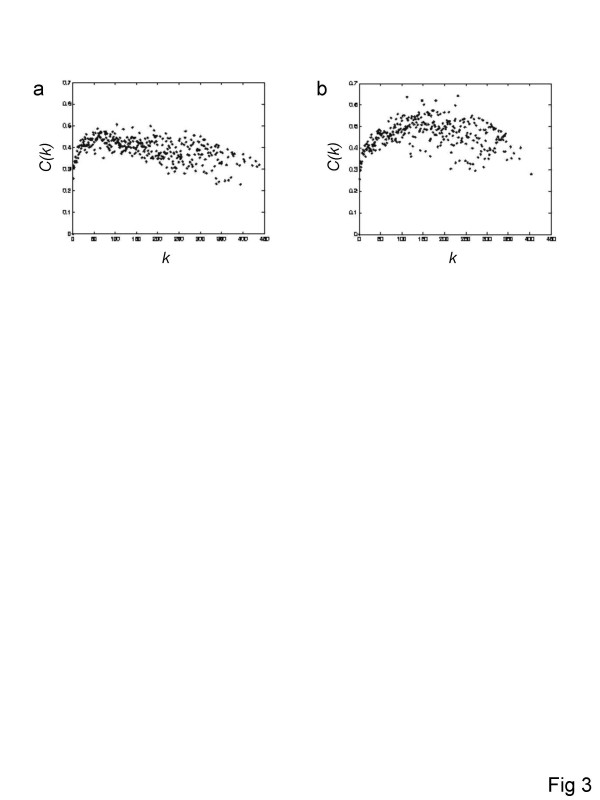
**Clustering coefficient against node degree *C(k) *distributions for human (a) and mouse (b) gene coexpression networks**. The degree *(k) *is shown on the x-axis and the average clustering coefficient <*C*> for all nodes with degree *k *is shown on the y-axis.

It has been shown that analysis of the plot of the clustering coefficient *C(k) *as a function of their degree *k*_*i *_can yield insight to the structure of the network. In particular, it has been reported that the *C(k) *distribution of networks with hierarchical structure follows a power-law, with a high interconnectivity among nodes of low degree that decreases as the degree increases [18]. The *C(k) *distribution of the human and mouse coexpression networks is more or less constant (Figures 3a and 3b) implying that these networks, most likely, do not exhibit a hierarchical structure. Slightly different trends were observed for the different distance or similarity measures (see Additional file 1; Supplementary Figure 4).

### Human-mouse intersection network

**Figure 4:**
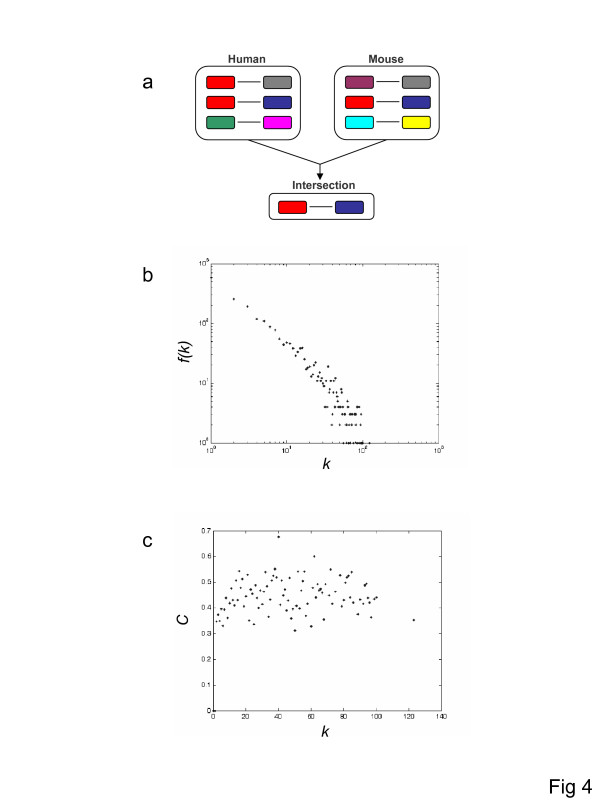
**Human-mouse conserved intersection network**. a) Procedure for computing the intersection network whereby conserved edges that link the corresponding orthologous genes in both species are preserved. b) Node degree *(k) *and c) clustering coefficient against node degree *C(k) *distributions for the intersection network.

As described above, the human and mouse gene coexpression networks are closely similar in terms of their global topological characteristics; they share similar node degree (*k*) distributions and *C(k) *distributions as well as similar average node degrees (<*k*>), clustering coefficients (<*C*>) and path lengths (<***l***>). We further sought to evaluate the similarity between the species-specific coexpression networks at a local level. There is as yet no general method for assessing local network similarity (or graph isomorphism). However, in the case of the human and mouse coexpression networks generated here, the use of orthologous gene pairs results in a one-to-one mapping between the nodes of the two networks. In this sense, the networks can be considered to be defined over the same set of nodes *N*, and thus can be directly compared by generating an intersection network. The human-mouse intersection network is defined as the network over the set of nodes *N *where there is a link between two nodes *i *and *j *if *i *and *j *denote two pairs of orthologous genes which are connected in both the human and the mouse networks (Figure 4a). Thus, the intersection network captures the coexpressed gene pairs conserved between human and mouse.

The global characteristics of the intersection network are shown in Figures 4b and 4c. The intersection network node degree and *C(k) *distributions are clearly similar to those of the species-specific networks as are the average clustering coefficient (<*C*> = 0.4006) and average path length (<***l***> = 6.89). The exponent that best approximates the power law of the node degree distribution is *α *= 1.34 when a line is fitted to the logarithmically binned distribution (see Additional file 1; Supplementary Figure 5) and *α *= 1.01 using the maximum likelihood method. Taken together, these findings indicate that the global structure of the species-specific coexpression networks is preserved in the intersection network. However, the most striking feature of the intersection network is the small fraction of genes (~29–31%) and edges (~7–8%) that are conserved between the human and mouse networks (Table 2). Accordingly, the average node degree is far lower (<*k*> = 11.57) in the intersection network than it is in each of the species-specific networks.

**Table 2:** Local conservation of the human-mouse intersection network

	**Intersection**^**1**^	**% Human**^**2**^	**% Human *N***^**3**^	**% Mouse**^**2**^	**% Mouse *N***^**3**^
Nodes	2,257	31.31	63.20	29.20	41.51
Edges	13,060	8.24	11.71	7.33	4.93

^1^Number of nodes and edges in the human-mouse intersection network

^2^Percentage of the nodes and edges conserved in the intersection network relative to the human and mouse networks

^3^Normalized percentage of the nodes and edges (see Additional file 1) conserved in the intersection network

Several other factors also point to the local level divergence of the human and mouse coexpression networks. When the degrees (*k*) of nodes present in both the human and mouse networks were arranged into species-specific degree sequence vectors, only relatively low, albeit statistically significant (given the large number of observations), correlation (*r *= 0.27, *P *= 9e-149) was seen between species. In other words, a highly connected node (hub) in the human coexpression network is not especially likely to be a hub in the mouse coexpression network and *vice versa*. In addition, the human and mouse coexpression *r*-values for shared edges are not correlated at all (*r *= 0.03). Finally, there is no correlation between the principal eigenvector values of the human and mouse networks (*r *= -0.03), indicating that the dense areas of the networks do not overlap. Thus, whereas the global topological properties of the species-specific networks are highly conserved, the local architectures that underlie these topologies, in terms of the identities of the coexpressed genes pairs, are highly divergent.

The low level of conservation seen for the local network structures was unexpected, particularly, in light of the close similarity of the global topological properties, and suggested substantial divergence of gene expression patterns between human and mouse orthologs. A series of controls were implemented to assess the meaning and robustness of these findings (see Additional file 1). These controls included comparison of networks constructed separately from experimental and biological replicate data sets, and analysis of network conservation for subsets of the data with different experimental variances. The results of these controls indicate that the majority of the local divergence between human and mouse coexpression networks does not result from experimental noise. In addition, lowering the *PCC *threshold used to define edges in the coexpression networks does not result in a substantial increase in the fraction of edges conserved between species (see Additional file 1; Supplementary Figure 9a).

The high divergence of coexpressed gene pairs between human and mouse detected here is consistent with previous studies that have shown substantial divergence of the expression profiles for human and mouse orthologs [5,6,21,22]. Indeed, when the expression profiles were directly compared for the 9,105 human-mouse orthologous gene pairs studied here, the average *PCC*, while positive, was fairly low and not statistically significant (average *PCC *= 0.22, Student's t = 1.15, df = 26, *P *= 0.26).

### Functional coherence of gene coexpression networks

**Figure 5:**
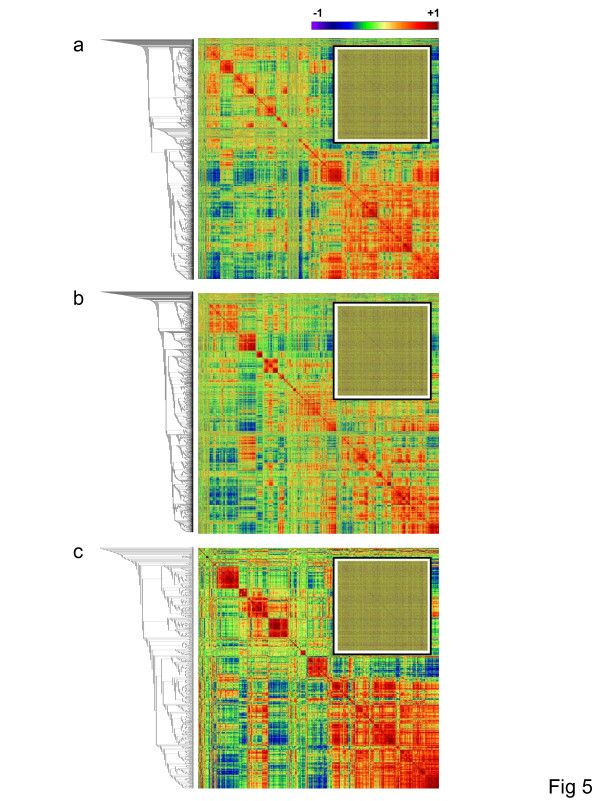
**GO similarity versus gene profile correlation matrix**. Genes are plotted along both axes of the matrices. Genes were clustered according to the pairwise similarity between their GO biological process annotation terms for the a) human-specific coexpression network, b) mouse-specific coexpression network and c) the human-mouse conserved intersection network. Pearson correlations (*r*) for all pairs of tissue-specific gene expression profiles are plotted according to the color bar. The inset of each plot shows a negative control where genes are randomly plotted, *i.e*. without regard to functional similarity, along the axes of the matrix.

The coexpression networks described here are analytical constructs that are intended to capture the complexity of the relationships among thousands of gene expression patterns. Given the significant rapidly evolving (and perhaps neutral) component in the evolution of these networks, it is not a trivial question whether or not (and to what extent) coexpressed gene pairs represent coregulated and/or functionally interacting genes. To assess the biological relevance of these networks, Gene Ontology (GO) functional annotations were mapped onto the network nodes and the functional affinities of linked genes were explored. The first question addressed was whether, and to what extent, coexpressed genes are functionally related. The structure of the GO graph can be exploited to derive measures of functional similarity between pairs of genes [23]. Pairwise similarities between biological process terms were computed for all pairs of network genes associated with GO annotations, and these functional similarity data were then used to cluster genes by the UPGMA method. The resulting lists of genes, ordered by function, were plotted on both axes of a matrix containing all pairwise gene expression profile correlations. When these correlations (*r*-values) are color coded, it allows for a visual inspection of the functional relationships, or lack thereof, among coexpressed genes (Figure 5). The functional coherence of the human-specific, mouse-specific, and intersection networks is clearly revealed by the off-diagonal block color-structure of plots (Figure 5a, 5b, and 5c, respectively). In each of these networks, there are numerous clusters of functionally related genes that are demonstrably enriched for coexpressed pairs. For comparison, the inset of each plot shows a negative control with genes ordered randomly along the matrix axes, and accordingly, no apparent block color-structure for the correlation values.

In addition to this visual evidence for the functional affinity of coexpressed gene pairs, genes linked in the coexpression networks were found to have significantly higher GO similarities, on average, than seen for all pairs of genes (Table 3). In addition, statistically significant positive correlations were detected between the pairwise coexpression *r*-values and GO similarity values for all three coexpression networks, indicating that more tightly coexpressed gene pairs tend to be more functionally related (Table 4). The correlation was significantly greater for the intersection network than for each of the species-specific networks.

**Table 3:** Average GO similarity for mammalian gene coexpression networks versus average GO similarity for all gene pairs

**Species**	***netGOavg***^**1**^	***allGOavg***^**2**^	***t***^**3**^	***P***^**4**^
Human	0.2637 ± 9.1e-4	0.1989 ± 4.9e-5	80.78	0
Mouse	0.2736 ± 8.9e-4	0.2150 ± 8.2e-5	75.23	0

^1^Average GO similarities for all pairs of genes connected by an edge in the coexpression network

^2^Average GO similarities all possible pairs among the 9,105 human-mouse orthologs

^3^Value of test statistic, *t *= (*μ1*-*μ2*)/(σd*sqrt(1/*n1*+1/*n2*)) where *μ *are the respective means and *σd *is the standard deviation of the difference

^4^Level of significance based on Students t-distribution with degrees of freedom = *n*1+*n*2-2

**Table 4:** Correlation (*r*) between pairwise GO similarity and pairwise gene expression profile *r*-values

**Network**	***r***^**1**^	***n***^**2**^	***t***^**3**^	***P***^**4**^
Human	0.1012	49303	22.59	2.2e-112
Mouse	0.0974	57685	23.50	1.4e-121
Intersection	0.1927	5370	14.39	4.4e-46

^1^Pearson correlation coefficient

^2^Number of gene pairs compared

^3^Value of test statistic, *t *= r*sqrt((*n*-2)/(1-*r*^2^))

^4^Level of significance based on Students *t*-distribution with degrees of freedom = *n*-2

**Figure 6:**
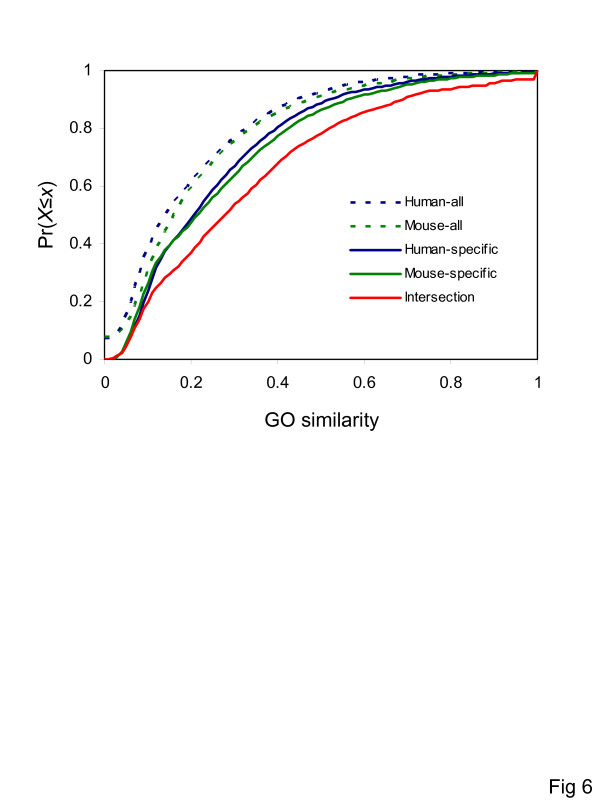
**GO biological process semantic similarity cumulative frequency distributions**. Distributions [*Pr(X*≤*x)*] of GO term similarities are shown for all human and mouse gene pairs, for pairs of genes linked in the species-specific coexpression networks and for pairs of genes linked in the conserved human-mouse intersection network.

Based on visual comparison of the off-diagonal color structure of the plots shown in Figure 5a,b versus Figure 5c, there appears to be a stronger relationship between function and coexpression for the genes that are found in the conserved human-mouse intersection network than for the human or mouse networks. This suggests that the expression patterns of gene pairs that are tightly functionally coupled are more prominently constrained by purifying selection than those of more loosely functionally associated genes. Statistical comparison of the species-specific versus intersection network supports this interpretation. Pairs of coexpressed genes in the intersection network are significantly more functionally similar, on average, than pairs of coexpressed genes in the species-specific networks (Table 5). A cumulative frequency distribution of GO similarities between pairs of mammalian genes clearly shows that genes linked in the intersection network are more functionally similar than genes linked in the species-specific portions of the network, which are in turn more similar than all pairs of genes irrespective of their expression patterns (Figure 6). Finally, there was a significantly stronger dependence between coexpression and function for gene pairs in the intersection network compared to the species-specific pairs as indicated by a comparison between expression profile correlations and GO functional similarity (Table 6).

**Table 5:** Average GO similarity for species-specific mammalian gene coexpression networks versus average GO similarity for the conserved human-mouse intersection network

**Network**	***GOavg***^**1**^	***t***^**2**^	***P***^**3**^
Human-specific	0.2556 ± 9.3e-4	25.65	3.3e-144
Mouse-specific	0.2678 ± 9.2e-4	20.31	2.2e-91
Intersection	0.3299 ± 3.4e-3		

^1^Average GO similarities for all pairs of genes connected by an edge in the coexpression network

^2^Value of the test statistic for the comparison between the species-specific network and the intersection network, *t *= (*μ1*-*μ2*)/(σd*sqrt(1/*n1*+1/*n2*)) where *μ *are the respective means and *σd *is the standard deviation of the difference

^3^Level of significance, *i.e*., probability that the distributions of GO similarity values for the intersection and species-specific networks are identical, based on Students t-distribution with degrees of freedom = *n*1+*n*2-2

**Table 6:** Correlation (*r*) between pairwise GO similarity and pairwise gene expression profile *r*-values

**Network**	***r***^**1**^	***n***^**2**^	***z***^**3**^	***P***^**4**^
Human-specific	0.0581	43933	9.47	0
Mouse-specific	0.0730	52315	8.51	0
Intersection	0.1927	5370		

^1^Pearson correlation coefficient

^2^Number of gene pairs compared

^3^Value of the test statistic for the comparison between the species-specific network and the intersection network, *z *= (*zf*1-*zf*2)/sqrt(1/(*n*1–3)+1/(*n*2–3)) where *zf *is the Fisher transform, *zf *= 1/2*ln((1+*r*)/(1-*r*))

^4^Level of significance, *i.e*., probability that the correlations in the intersection and species-specific networks are indistinguishable, based on normal distribution with infinite degrees of freedom

### Network clusters and biological function

**Figure 7:**
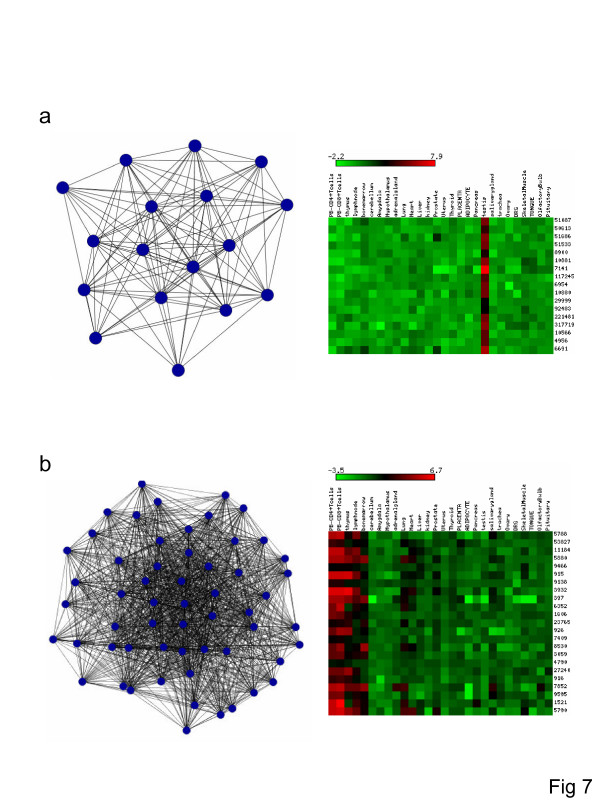
**Clusters of tightly coexpressed and functionally coherent genes**. Examples of clusters involved in spermatogenesis (a) and host immune response (b) are shown along with their tissue-specific expression patterns.

The mammalian coexpression networks analyzed here are tightly clustered, as indicated by the high average clustering coefficients (Table 1), and display modular, albeit not necessarily hierarchical, structure (see Additional file 1; Supplementary Figure 6), (Figure 3 and Figure 4c). In light of the presence of compact network substructures, further functional interrogation was performed by decomposing the networks into tightly linked clusters of genes. The genes in these clusters were then evaluated for the presence of statistically overrepresented GO terms, which would indicate functional coherence for the respective group of genes. In a number of cases, there are striking relationships between network substructure, gene function and coexpression. A detailed table showing resolved network clusters, overrepresented GO terms and gene ids along with their expression patterns is presented online [24]. Two of the most prevalent functional classes that show clear function-expression coherence are genes involved in sexual reproduction and host immune response. Examples of two such clusters are shown in Figure 7. This observation is notable because genes of these two functional classes are also prone to evolve under the influence of positive, diversifying selection [25,26]. This is thought to be due to sexual selection, in the case of reproduction related genes [27], and to evolutionary arms race between hosts and their pathogens for immune response genes [28]. It might prove to be the case that changes in gene expression patterns for such genes also have pronounced evolutionary significance.

Consistent with the apparent increased functional coherence of the intersection network, the correspondence between network clusters, GO term overrepresentation and expression patterns is significantly more pronounced for the conserved intersection network than for the human and mouse species-specific networks. Thus, 38% of clustered genes from the intersection network mapped to overrepresented GO terms compared to 13% of human-specific (χ^2 ^= 85.1, *P *= 2.8e-20) and 18% of mouse-specific network genes (χ^2 ^= 43.0, *P *= 5.5e-11).

## Conclusion

### General significance of coexpression network structure

The global topological properties of the human and mouse gene coexpression networks studied here are very similar but the specific architectures that underlie these properties are drastically different. In other words, the actual pairs of orthologous genes that are found to be coexpressed in the different species are highly divergent, although we did detect a substantial conserved component of the co-expression network. The discordance between evolutionary conservation at distinct levels of network organization has implications for understanding the general significance of the topological properties of networks that represent various complex systems.

The last few years have seen an explosion of studies on various kinds of biological and non-biological networks [29,30]. A central theme for much of this work has been the striking unity of the topological properties of networks representing very different complex systems, from biological (*e.g*., metabolic and protein interaction) networks to non-biological ones, such as social interaction networks and the world-wide-web. Almost all these complex networks show evidence of both scale-free [31] and small world [32] properties. In other words, the network node-degree distributions fit power laws and the diameter of the networks, in terms of the average number of links between two nodes in the network, stays small despite increases in network size. These observations have led to the hope that the network perspective might 'revolutionize our view of biology' [18]. This hope is based on the idea that similar network properties are a result of universal laws that govern evolution and architecture of complex systems. As such, the comprehension of these basic laws, or simple principles, has the potential to yield unprecedented insight into biological organization and evolution. Implicit in this stance is the emphasis on a systems-level view of biology, which considers ensembles of interacting parts (genes, proteins etc.) as opposed to individual actors alone.

While this optimistic perspective on the biological significance of network topologies generated considerable excitement in some quarters, it has not gone unchallenged. A more guarded view of these findings holds that the conserved global topological properties of biological networks might actually reveal little or nothing about the evolutionary mechanisms that gave rise to them or the particular nature of their organization [33-36]. Instead, the relevant architectural features of the individual networks could be quite specific and determined by the functional constraints on the particular system. This world-view stresses the anecdotal nature of biological sciences, placing the focus back on the nature of the individual genes, proteins and/or systems under consideration, and eschews the search for universal laws. Based on the results obtained here, it would seem that mammalian gene expression evolves more in accordance with the latter, more cautious view on the significance, or lack thereof, of conserved network properties. In the case of gene expression, the highly conserved global network properties belie highly divergent local structures that result from the rapid evolution of gene expression patterns. Thus, the architecture of the coexpression networks is highly species-specific and the conservation of the global network properties occurs despite, not because of, extensive evolutionary changes in gene expression.

Accordingly, at least in the case of gene expression divergence, the biological relevance of the global network topological properties appears questionable. Of course, this does not prevent network analysis from being a powerful approach, possibly, the most appropriate one for the quantitative study of complex systems made up of numerous interacting parts. It is also worth noting that coexpression networks built from randomly permuted expression vectors differ from the observed networks in not containing high-degree nodes (hubs) and thus cannot be claimed to possess scale-free properties with respect to their node degree distributions (data not shown). Thus, some biological features of expression patterns that yield the observed node-degree distributions in coexpression networks might exist; identifying such features could be important for understanding evolution of gene expression.

With regard to the more specific aspects of this work, the conservation of a small but substantial component of the coexpression network indicates that, the rapid evolution notwithstanding, the network evolves under the constraints of purifying selection. The biological significance of the rapid interspecies divergence of coexpression networks remains an open problem [4-6,21,37,38]. It is yet unclear how much of this divergence is neutral, biologically irrelevant noise and how much is functional divergence driven by positive selection and defining, in part, salient differences in the biology of the respective organisms. Addressing these questions is an important goal for future network studies.

## Methods

### Orthologous gene expression

Gene expression data, based on Affymetrix microarray experiments, for human, and mouse are obtained from the mammalian gene expression atlas [17]. These expression data were retrieved from the UCSC Genome Browser [39]. Affymetrix probe identifiers (ids) were mapped to human and mouse genomic loci using UCSC Genome Browser and NCBI annotations as shown below:

Affymetrix probe id → GenBank accession → RefSeq accession → NCBI Locus id

Only affymetrix probes that map to unique genomic loci were considered for further analysis. When loci were found to be covered by multiple probes, the probe yielding the highest overall expression level was used in subsequent analyses.

In order to directly compare gene coexpression networks of different species, a set of orthologous genes expressed over a set of common tissue samples was analyzed. 9,105 orthologous human-mouse genes pairs were identified, using reciprocal best BLASTP hits [40], along with 28 common tissues with expression data for both human and mouse. For each gene, for each tissue, there were two replicate measurements. The average of these two values was taken to produce a 9105 × 28 matrix of real values. This matrix was further normalized as follows. For each gene, the median of the expression values of the gene across all tissues was computed and the entries of the corresponding matrix row were normalized with this value. These values were then log2 normalized resulting in a set of values with median zero.

Vectors of normalized tissue-specific expression levels were compared using a number of different measures: Euclidean distance, Manhattan distance, Jensen-Shannon entropy, dot-product, cosine similarity and Pearson correlation coefficient. Results reported in the body of the manuscript are for networks constructed using the Pearson correlation coefficient (*PCC*), and a discussion of results based on other measures is included in the Supplementary Information section (see Additional file 1).

### Network analysis

All-against-all gene expression profile comparisons for the human and mouse matrices (9,105 × 28) were used to generate species-specific coexpression networks. Network nodes correspond to genes and gene pairs with *PCC r*≥0.7 were linked by and edge. Networks' topological properties were analyzed using MATLAB^®^. For each network the number of nodes and number of edges was simply counted. The average degree <*k*> was calculated as the average number of connections per node. The average clustering coefficient <*C*> was calculated as the average clustering coefficient of all nodes with at least two neighbors using the formula: *Ci *= 2*n*_*i*_/*ki*(*ki*-1), where *n*_*i *_is the number of observed links connecting the *k*_*i *_neighbors of node *i *and *k*_*i*_(*k*_*i*_-1)/2 is the total number of possible links. The average path length (<***l***>) was calculated as the average shortest path, or the smallest number of edges needed to connect two nodes, between any two reachable nodes in the network. Node degree distributions were plotted with the degree *(k) *on the x-axis and the number of nodes with this degree *f(k) *on the y-axis. Clustering coefficient against node degree *C(k) *distributions were plotted with the degree *(k) *on the x-axis and the average clustering coefficient <*C*> for all nodes with degree *k *on the y-axis. Species-specific networks were compared to derive a conserved intersection network containing only edges that connect the same orthologous genes (Figure 4a), and the network properties of the intersection network were calculated. Controls for experimental variance were performed by constructing two replicate-specific networks for human and mouse respectively and then computing the species-specific replicate intersection networks. A normalized intersection network was calculated by comparing the two species-specific replicate intersection networks. A control for experimental and biological variance was conducted by comparing mouse expression data from Novartis [17] with and independently obtained mouse expression data set [41].

### Functional analysis

Network visualization and functional analysis was done using Cytoscape [42]. Networks were partitioned into tightly linked clusters of genes using MCODE [43]. Genes in the networks were functionally categorized using their Gene Ontology (GO) biological process annotation terms [44]. Overrepresented GO terms were identified with BINGO [45] by comparing the relative frequencies of GO terms in specific clusters with the frequencies of randomly selected GO-terms. The Hypergeometric test was used to do this with the Benjamini and Hochberg false discovery rate correction for multiple tests and a *P*-value threshold of 0.001. Pairwise similarities between gene GO terms were measured using the semantic similarity method, which computes the relative distance between any two terms along the GO-graph [23].

## Authors' contributions

PT performed the network analyses and helped to draft the manuscript. LMR participated in the design of the study and performed network, expression and GO analyses. OB calculated the between gene GO annotation similarities. EVK participated in the design of the study and contributed to the writing of the manuscript. IKJ participated in the design of the study, performed network and expression analyses and contributed to the writing of the manuscript. All authors read and approved the final manuscript.

## Supplementary Material

Additional File 1 Further information on i-coexpression distance measures, ii-global network characteristics and iii-node degree distributions. Click here for file

## References

[B1] Britten RJ, Davidson EH (1969). Gene regulation for higher cells: a theory. Science.

[B2] Britten RJ, Davidson EH (1971). Repetitive and non-repetitive DNA sequences and a speculation on the origins of evolutionary novelty. Q Rev Biol.

[B3] King MC, Wilson AC (1975). Evolution at two levels in humans and chimpanzees. Science.

[B4] Khaitovich P, Weiss G, Lachmann M, Hellmann I, Enard W, Muetzel B, Wirkner U, Ansorge W, Paabo S (2004). A neutral model of transcriptome evolution. PLoS Biol.

[B5] Yanai I, Graur D, Ophir R (2004). Incongruent expression profiles between human and mouse orthologous genes suggest widespread neutral evolution of transcription control. Omics.

[B6] Jordan IK, Marino-Ramirez L, Koonin EV (2005). Evolutionary significance of gene expression divergence. Gene.

[B7] Khaitovich P, Hellmann I, Enard W, Nowick K, Leinweber M, Franz H, Weiss G, Lachmann M, Paabo S (2005). Parallel patterns of evolution in the genomes and transcriptomes of humans and chimpanzees. Science.

[B8] Luscombe NM, Babu MM, Yu H, Snyder M, Teichmann SA, Gerstein M (2004). Genomic analysis of regulatory network dynamics reveals large topological changes. Nature.

[B9] Gu Z, Nicolae D, Lu HH, Li WH (2002). Rapid divergence in expression between duplicate genes inferred from microarray data. Trends Genet.

[B10] Makova KD, Li WH (2003). Divergence in the spatial pattern of gene expression between human duplicate genes. Genome Res.

[B11] Madan Babu M, Teichmann SA, Aravind L (2006). Evolutionary Dynamics of Prokaryotic Transcriptional Regulatory Networks. J Mol Biol.

[B12] Ge H, Liu Z, Church GM, Vidal M (2001). Correlation between transcriptome and interactome mapping data from Saccharomyces cerevisiae. Nat Genet.

[B13] Wuchty S, Barabasi AL, Ferdig MT (2006). Stable evolutionary signal in a Yeast protein interaction network. BMC Evol Biol.

[B14] Fraser HB, Hirsh AE, Wall DP, Eisen MB (2004). Coevolution of gene expression among interacting proteins. Proc Natl Acad Sci U S A.

[B15] Wolfe CJ, Kohane IS, Butte AJ (2005). Systematic survey reveals general applicability of "guilt-by-association" within gene coexpression networks. BMC Bioinformatics.

[B16] Ihmels J, Bergmann S, Gerami-Nejad M, Yanai I, McClellan M, Berman J, Barkai N (2005). Rewiring of the yeast transcriptional network through the evolution of motif usage. Science.

[B17] Su AI, Wiltshire T, Batalov S, Lapp H, Ching KA, Block D, Zhang J, Soden R, Hayakawa M, Kreiman G, Cooke MP, Walker JR, Hogenesch JB (2004). A gene atlas of the mouse and human protein-encoding transcriptomes. Proc Natl Acad Sci U S A.

[B18] Barabasi AL, Oltvai ZN (2004). Network biology: understanding the cell's functional organization. Nat Rev Genet.

[B19] Dorogovtsev SN, Mendes JFF (2003). Evolution of networks: from biological networks to the internet and WWW.

[B20] Koonin EV, Wolf YI, Karev GP (2006). Power laws, scale-free networks and genome biology.

[B21] Jordan IK, Marino-Ramirez L, Wolf YI, Koonin EV (2004). Conservation and coevolution in the scale-free human gene coexpression network. Mol Biol Evol.

[B22] Su AI, Cooke MP, Ching KA, Hakak Y, Walker JR, Wiltshire T, Orth AP, Vega RG, Sapinoso LM, Moqrich A, Patapoutian A, Hampton GM, Schultz PG, Hogenesch JB (2002). Large-scale analysis of the human and mouse transcriptomes. Proc Natl Acad Sci U S A.

[B23] Lord PW, Stevens RD, Brass A, Goble CA (2003). Investigating semantic similarity measures across the Gene Ontology: the relationship between sequence and annotation. Bioinformatics.

[B24] Fay JC, Wu CI (2001). The neutral theory in the genomic era. Curr Opin Genet Dev.

[B25] Yang Z, Bielawski JP (2000). Statistical methods for detecting molecular adaptation. Trends  Ecol Evol.

[B26] Wyckoff GJ, Wang W, Wu CI (2000). Rapid evolution of male reproductive genes in the descent of man. Nature.

[B27] Bergelson J, Kreitman M, Stahl EA, Tian D (2001). Evolutionary dynamics of plant R-genes. Science.

[B28] Barabasi AL (2002). Linked: how everything is connected to everything else and what it means.

[B29] Watts DJ (2003). Six degrees: the science of a connected age.

[B30] Barabasi AL, Albert R (1999). Emergence of scaling in random networks. Science.

[B31] Watts DJ, Strogatz SH (1998). Collective dynamics of 'small-world' networks. Nature.

[B32] Gisiger T (2001). Scale invariance in biology: coincidence or footprint of a universal mechanism?. Biol Rev Camb Philos Soc.

[B33] Keller EF (2005). Revisiting "scale-free" networks. Bioessays.

[B34] Newman MEJ (2005). Power laws, Pareto distributions and Zipf's law. Contemporary Physics.

[B35] Wolf YI, Karev G, Koonin EV (2002). Scale-free networks in biology: new insights into the fundamentals of evolution?. Bioessays.

[B36] Liao BY, Zhang J (2006). Low Rates of Expression-Profile Divergence in Highly-Expressed Genes and Tissue-Specific Genes During Mammalian Evolution. Mol Biol Evol.

[B37] Liao BY, Zhang J (2006). Evolutionary conservation of expression profiles between human and mouse orthologous genes. Mol Biol Evol.

[B38] Karolchik D, Baertsch R, Diekhans M, Furey TS, Hinrichs A, Lu YT, Roskin KM, Schwartz M, Sugnet CW, Thomas DJ, Weber RJ, Haussler D, Kent WJ (2003). The UCSC Genome Browser Database. Nucleic Acids Res.

[B39] Altschul SF, Madden TL, Schaffer AA, Zhang J, Zhang Z, Miller W, Lipman DJ (1997). Gapped BLAST and PSI-BLAST: a new generation of protein database search programs. Nucleic Acids Res.

[B40] Zhang W, Morris QD, Chang R, Shai O, Bakowski MA, Mitsakakis N, Mohammad N, Robinson MD, Zirngibl R, Somogyi E, Laurin N, Eftekharpour E, Sat E, Grigull J, Pan Q, Peng WT, Krogan N, Greenblatt J, Fehlings M, van der Kooy D, Aubin J, Bruneau BG, Rossant J, Blencowe BJ, Frey BJ, Hughes TR (2004). The functional landscape of mouse gene expression. J Biol.

[B41] Shannon P, Markiel A, Ozier O, Baliga NS, Wang JT, Ramage D, Amin N, Schwikowski B, Ideker T (2003). Cytoscape: a software environment for integrated models of biomolecular interaction networks. Genome Res.

[B42] Bader GD, Hogue CW (2003). An automated method for finding molecular complexes in large protein interaction networks. BMC Bioinformatics.

[B43] Ashburner M, Ball CA, Blake JA, Botstein D, Butler H, Cherry JM, Davis AP, Dolinski K, Dwight SS, Eppig JT, Harris MA, Hill DP, Issel-Tarver L, Kasarskis A, Lewis S, Matese JC, Richardson JE, Ringwald M, Rubin GM, Sherlock G (2000). Gene ontology: tool for the unification of biology. The Gene Ontology Consortium. Nat Genet.

[B44] Maere S, Heymans K, Kuiper M (2005). BiNGO: a Cytoscape plugin to assess overrepresentation of gene ontology categories in biological networks. Bioinformatics.

[B45] Babu MM, Grant RP (2004). Introduction to microarray data analysis. Computational Genomics: Theory and Application.

