# Supplementary material for: Global similarity and local divergence in human and mouse gene co-expression networks
Source: BMC Evol Biol. 2006 Sep 12;6:70. doi: 10.1186/1471-2148-6-70 (PMC1601971; doi:10.1186/1471-2148-6-70)
Supplement: Additional File 1 — Further information on i-coexpression distance measures, ii-global network characteristics and iii-node degree distributions. [file 1471-2148-6-70-S1.pdf]

Supplementary Information for:

Global similarity and local divergence in human and mouse gene co-expression networks

Panayiotis Tsaparas[1], Leonardo Mariño-Ramírez[2], Olivier Bodenreider[3], Eugene V. Koonin[2]*, and I. King Jordan[4]

[1]Basic Research Unit, Helsinki Institute for Information Technology, University of Helsinki, Helsinki, Finland

[2]National Center for Biotechnology Information, National Institutes of Health, Bethesda, MD 20894, USA

[3]National Library of Medicine, National Institutes of Health, Bethesda, MD 20894, USA

[4]School of Biology, Georgia Institute of Technology, Atlanta, GA 30332, USA

*Corresponding author

Email addresses:

PT: tsaparas@cs.helsinki.fi
LMR: marino@ncbi.nlm.nih.gov
OB: olivier@nlm.nih.gov
EVK: koonin@ncbi.nlm.nih.gov
IKJ: king.jordan@biology.gatech.edu

Supplementary Results

Coexpression distance measures

A number of different metrics were used to measure the similarity (distance) between vectors of tissue-specific expression levels: Euclidean distance, Manhattan distance, Jensen-Shannon divergence, cosine similarity, Pearson correlation coefficient and dot-product.

The Euclidean and Manhattan distances both measure the distance between vectors in geometric space. The Euclidean distance (*dE*) between two vectors $x=(x_1 \ldots x_n)$ and $y=(y_1 \ldots y_n)$ is computed as: $dE=\left(\sum_{i=1}^{n}(x_i - y_i)^2\right)^{1/2}$. The Manhattan distance (*dM*) is computed as: $dM=\sum_{i=1}^{n}|x_i - y_i|$. The Jensen-Shannon divergence [1] is an information theoretic measure. To take this measure, expression values for each profile were normalized so that they sum to 1. In this way, we deal with one unit of expression distributed among the 28 tissues, *i.e.* an expression probability distribution. The Jensen-Shannon measure gives a way of comparing pairs of probability distributions by taking the average of the two distributions and measuring the relative entropy between the two observed distributions and the average distribution. Cosine similarity measures the cosine of the angle formed by the two vectors. The Pearson correlation coefficient is a measure of how well a linear equation describes the relation between the two vectors x and y. The dot-product of two vectors $(x \cdot y)$ is the sum of the products of the individual coordinates and is measured as: $(x \cdot y)=\sum_{i=1}^{n} x_i y_i = \|x\|\|y\|\cos(x, y)$. As can be seen from the equation, the dot-product can be computed as a weighted version of the cosine similarity.

The relationships between these methods, with respect to how they compare gene expression profiles, were computed by comparing gene coexpression networks constructed using the different methods. Coexpression networks were built using each distance (similarity) measure using thresholds for linking coexpressed genes that resulted in networks with comparable numbers of edges. Then, for each pair of methods, the intersection (in terms of edges) of their two networks was computed and normalized by the union of the two networks. This value was taken as the similarity (s) between the methods. All pairwise similarities were converted to distances (d=1-s), and the pairwise distance matrix was used to cluster the different methods (Supplementary Figure 1). The methods fall into two distinct clusters: distance and correlation. Obviously, the Euclidean and Manhattan distances are quite similar so it is not surprising that they group together. However, the grouping of the Jensen-Shannon information theoretic measure of divergence with these two measures was less expected. We currently do not have any explanation for this. The close relationship between cosine similarity and Pearson correlation coefficient can also be expected since the Pearson correlation coefficient is identical to cosine similarity when the mean of the vectors is equal to zero. The gene expression profile vectors used here are normalized by the median so these two measures are quite similar. Apparently, these measures can be classified as i-distance methods (Euclidean distance, Manhattan distance, Jensen-Shannon divergence) or ii-correlation methods (cosine similarity and Pearson correlation coefficient). The dot-product is an analytically distinct from these two classes as shown by its separation from the other methods (Supplementary Figure 1). This seems somewhat unexpected because the dot-product is analytically related to both the cosine similarity (see equation above) and the Pearson correlation coefficient. The main difference is that the dot-product

weighs the distance by the length of the vectors, which in the case of the data analyzed here corresponds to the level of gene expression. This underscores the prominent effect of expression levels on comparisons between expression profiles, and confirms the importance of using measures that control for this, such as the Pearson correlation coefficient, when considering relative levels of expression across tissues.

Global network characteristics

Global characteristics of human and mouse gene coexpression networks were calculated using six different distance (similarity) measures: Pearson correlation coefficient, cosine similarity, Euclidean distance, Manhattan distance, dot-product and Jensen-Shannon divergence. Global characteristics are shown in Supplementary Table 1 as well as Supplementary Figures 3 and 4.

Node degree distributions

The node degree distributions for the human and mouse gene coexpression networks seem to follow a power-law where the probability that a randomly chosen node has degree *k*, is $\Pr[K = k] \propto k^{-\alpha}$. However, there appears to be an exponential drop-off in the tail of the distributions, so they may not strictly correspond to power-laws.

Another method for estimating power-laws is logarithmic binning where the nodes are binned together depending on their degree. The bins are selected such that they grow exponentially, i.e., nodes whose degree falls in the intervals [1,2), [2,4), [4,8), …, $[2^k, 2^{k+1})$ are binned together. The numbers of nodes that fall within each bin are counted and normalized by the size of the bin (Supplementary Figure 2a and 2b). The log-log plot of the binned distribution gives a much better estimate of the power-

law distribution by eliminating the noise that is usually introduced in the tail of the distribution. The points seem to fall on a straight line, with the exception of the last point. The least squares approximation gives exponents of α=1.13 for the human network and α=1.11 for the mouse network. It should be noted that the last point of the plot cannot be disregarded as it comprises a bin of size equal to the size of all previous bins together, even if it does not include many data points; thus, the position of this last point is diagnostic of the behavior of the distribution tail.

The shape of the cumulative distribution $\Pr[D \geq d]$ of the network degrees shows to worst fit to a power-law (Supplementary Figure 2c and 2d). If the degree distribution follows a power law with exponent α, then the cumulative distribution should follow a power law with exponent α-1, that is, $\Pr[D \geq d] \propto d^{-\alpha+1}$. Obviously, that is not the case here and the cumulative distribution does not seem to be well approximated by a straight line.

Finally, a non-standard maximum likelihood method for computing the exponent of a power law distribution [2] gives α=1.38 for the human network, and α=1.36 for the mouse network.

Network comparison controls

A series of control analyses were performed to evaluate the possibility that the apparent high local divergence of the two networks might stem from a high level of experimental noise. The first control tests against the null hypothesis that the number of network edges found to be conserved between species could be expected by chance alone. Randomly rewired networks were used to approximate the null distribution for the number of conserved edges in the intersection network. The approach used to randomly rewire networks involves swapping edges among pairs of connected nodes

[3]. For instance, two edges are chosen, (x,y) and (z,w), such that x is not linked to w and z is not linked to y, and the existing edges are then exchanged yielding the new pairs (x,w) and (z,y). This approach is conservative in the sense that it ensures that the node degrees are preserved in the rewired networks. Supplementary Figure 7a shows a comparison of the number of edges shared by 1,000 randomly rewired human and mouse networks (μ=2,277.5, σ=44.95) with the number of edges actually observed in the human-mouse intersection network (13,060). Clearly, there are far more edges conserved between the human and mouse coexpression networks than expected by chance alone (Z=240, P=0). This indicates that, despite the high divergence between the species-specific networks, there is substantial conservation of the gene co-expression network structure between human and mouse, presumably, because evolution of gene expression is, to some extent, constrained by purifying selection.

Another set of controls was implemented to directly evaluate the effect of the quality and source of the expression data on the local network structure conservation between species. These controls took advantage of the fact that two replicate microarray experiments were performed for every tissue sample in the Novartis mammalian gene expression atlas (GNF SymAtlas) data set. In the first of these data quality controls, the expression data sets were partitioned into four slices of increasing between-replicate variance. For tissue-specific expression profiles, the coefficient of variance was computed for each pair of two tissue-specific replicate experiments, summed across all 28 tissues and averaged for human and mouse orthologs: $\frac{1}{2}\sum_{human}^{mouse}\sum_{i=1}^{28}\frac{\sigma}{\mu}$, where σ is the standard deviation and μ is mean of the two expression level measurements. Intersection networks were computed for these variance quartiles and the percentage of conserved edges were observed for each. There is

indeed a trend whereby the percentage of conserved edges decreases as the experimental variance increases (Supplementary Figure 7b). However, the magnitude of the effect is quite small and not nearly enough to explain the observed low level of between species conservation. In addition, the trend line that fits this data (y=-0.367) was not statistically significant when the residuals were evaluated using ANOVA, F=0.19, df=2, P=0.20, further underscoring that experimental variance alone cannot explain the low between species conservation.

Experimental replicates were also used to compute two replicate-specific data sets for each species, and then between-replicate intersection networks were built from these data. These experimental replicates measure variance in the RNA-isolation and hybridization processes. The percentage of conserved nodes and edges is far greater for the replicate intersection networks than for the between-species intersection network (Supplementary Figure 8a and 8b), further confirming that the divergence between species is not due primarily to experimental noise. The two species-specific replicate intersection networks were then compared to re-compute a normalized human-mouse intersection network that accounts for the loss of information on local network structure conservation caused by experimental noise. Although the normalized intersection network had a substantially greater proportion of conserved nodes, the fraction of conserved edges increased only slightly with respect to human and decreased slightly relative to the mouse (Table 2). This difference can be attributed to the fact that the mouse data show less between-replicate variance and as a result have a greater fraction of edges in the mouse-specific replicate intersection network.

Finally, a control that combined both experimental and actual biological variance was conducted using an independent microarray survey of mouse gene

expression levels [4], hereinafter referred to as the Toronto data set. When the Toronto data was compared to the GNF SymAtlas, ~61% of the nodes and 15% of the edges were conserved between the two sets. While the combination of the two sources of noise led to a substantial loss of coexpression signal, the level of conservation remained ~2x as high as seen between species for both nodes and edges.

In addition to the controls described above for experimental and biological variance, a series of PCC thresholds were used to evaluate the effect of the similarity measure strength on the level of between species conservation. We built human and mouse networks using PCC thresholds of 0.5, 0.6, 0.7, 0.8 and 0.9. The percentage of edges that are conserved in the intersection network increases as the PCC threshold is lowered (Supplementary Figure 9a). However, even at a very permissive r-value threshold of 0.5, which results in an order of magnitude increase in the number of observed co-expressed gene pairs, the fraction of conserved edges is still small (~14%). Interestingly, the relationship between the number of observed intersection edges, relative to the maximum possible number of such edges given the number of conserved nodes, and the PCC threshold is non-linear and forms a U-shaped distribution (Supplementary Figure 9b). At high r-values (≥0.9) the number of nodes (n) in the human and mouse coexpression networks is low, and thus the possible number of conserved edges in the intersection network [n(n-1)/2] is low as well. So, while the absolute number and percentage of conserved edges at this threshold is low, it actually represents a large fraction of the possible number of conserved edges. In other words, if a gene is coexpressed at r≥0.9 with any other gene in one of the species networks, it is likely to be involved in the same coexpression relationship in the other species network. As the r-value threshold drops (0.8-0.6) this effect disappears. This is because at these values more and more genes are involved in

coexpression relationships in either species network, but these coexpression relationships are not so strong as to guarantee that they are present in both networks. Then as the r-value drops even more (0.5), the number of nodes in both species networks, and accordingly the number of possible conserved edges, becomes saturated (i.e. reaches the total number orthologs analyzed-9,105) while the number of interactions continues to increase. Thus the fraction of conserved edges starts to increase again and reaches levels comparable to what was seen for the very conserved interactions at r≥0.9. This trend can be taken to suggest that the r-value threshold of 0.7 is close to optimal for defining coexpression relationships.

Supplementary References

1. Cover TM, Thomas JA: *Elements of Information Theory*. Wiley-Interscience; 1991.

2. Newman MEJ: **Power laws, Pareto distributions and Zipf's law.** *Contemporary Physics* 2005, **46:**323-351.

3. Milo R, Shen-Orr S, Itzkovitz S, Kashtan N, Chklovskii D, Alon U: **Network motifs: simple building blocks of complex networks.** *Science* 2002, **298:**824-827.

4. Zhang W, Morris QD, Chang R, Shai O, Bakowski MA, Mitsakakis N, Mohammad N, Robinson MD, Zirngibl R, Somogyi E, et al: **The functional landscape of mouse gene expression.** *J Biol* 2004, **3:**21.

5. Chakrabarti D: **AutoPart: Parameter-Free Graph Partitioning and Outlier Detection. .** In *European Conference on Principles and Practice of Knowledge Discovery in Databases; Pisa*. Springer; 2004

Supplementary Figures

Supplementary Figure 1 - Relationships among the different distance (similarity) measures

Gene coexpression networks were built using the six different measures of distance (similarity) between gene expression profile vectors. Measures were compared by taking the pairwise intersection of network edges normalized by the union of edges.

Supplementary Figure 2 - Node degree (*k*) distributions for human and mouse gene coexpression networks

Logarithmic binning where *k* falls into the intervals [1,2), [2,4), [4,8), …, [2^k,2k+1) for human (a) and mouse (b). Cumulative frequency distributions showing f[K≥k] for human (c) and mouse (d).

Supplementary Figure 3 - Node degree – *f(k)* × *k* – distributions for human and mouse gene coexpression networks

All distributions are plotted in $\log_{10}$-$\log_{10}$ scale. Distributions are shown for all six distance (similarity) measures used.

Supplementary Figure 4 - Clustering coefficient against node degree – *C(k)* × *k* – distributions for human and mouse gene coexpression networks

The degree *(k)* is shown on the x-axis and the average clustering coefficient <*C*> for all nodes with degree *k* is shown on the y-axis. Distributions are shown for all six distance (similarity) measures used.

Supplementary Figure 5 - Node degree (*k*) distributions for the conserved human-mouse intersection network

a) Logarithmic binning where *k* falls into the intervals [1,2), [2,4), [4,8), …, $[2^k,2^{k+1})$.

b) Cumulative frequency distributions showing $f[K \geq k]$.

Supplementary Figure 6 - Clustering of human (a) and mouse (b) gene coexpression networks

Clustering was done using the Autopart algorithm [5]. Genes are arranged along the axes according to network specific clusters. Dots correspond to edges between linked (coexpressed) genes. Blue dots are species-specific edges and red dots are edges found in the conserved human-mouse intersection network. Network modularity is revealed by the discrete block-diagonal structure of the dots.

Supplementary Figure 7 - Network comparison controls

a) Number of conserved edges for 1,000 comparisons of randomly rewired networks versus observed number of conserved edges between the human and mouse gene coexpression networks. b) Percentage of conserved edges for networks built independently four quartiles of increasing between replicate variance. Trend line fit to the data (y=-0.367) is shown.

Supplementary Figure 8 – Replicate network comparison controls

The percentage of intersection nodes (c) and edges (d) is shown for various replicate network comparisons. Percentages are shown relative to each of the networks (network1 & network 2) compared.

Supplementary Figure 9 – PCC threshold network comparison controls.

a) The percentage of intersection edges (y-axis) relative to the human (blue) and mouse (red) networks is shown for different Pearson correlation coefficient thresholds (x-axis). b) The percentage of intersection edges relative to the maximum number of possible edges given the number of shared nodes.

Tables

Supplementary Table 1 - Global characteristics of the coexpression networks

	PCC[1]	Cosine[1]	Euclidean[1]	Manhattan[1]	Dot-product[1]	JS[1]
**Human**						
**Nodes**[2]	7,208	7,031	3,436	3,694	4,922	4,017
**Edges**[3]	158,418	142,126	152,491	159,767	149,279	145,934
<***k***>[4]	21.98	20.21	44.38	43.25	30.33	36.33
<***C***>[5]	0.3744	0.3759	0.5530	0.5231	0.7828	0.5331
<***ℓ***>[6]	4.75	4.84	3.12	3.04	2.74	3.40
**Mouse**						
**Nodes**[2]	7,730	7,591	3,176	3,363	4,722	3,698
**Edges**[3]	178,166	170,046	175,625	194,062	211,097	195,000
<***k***>[4]	23.05	22.40	55.30	57.70	44.70	52.73
<***C***>[5]	0.4003	0.4089	0.5578	0.5409	0.8084	0.5533
<***ℓ***>[6]	4.80	4.87	3.09	2.94	2.67	3.50

[1]Distance (similarity) measure used: PCC=Pearson correlation coefficient, JS=Jensen-Shannon divergence
[2]Number of genes (nodes) in the network – *i.e.* nodes with one or more edges
[3]Number of coexpressed gene pairs (edges) in the network
[4]Average degree (*k*), number of edges shared with other nodes, per node
[5]Average clustering coefficient (*C*) per node
[6]Average shortest path length (*ℓ*) between any two nodes in the network

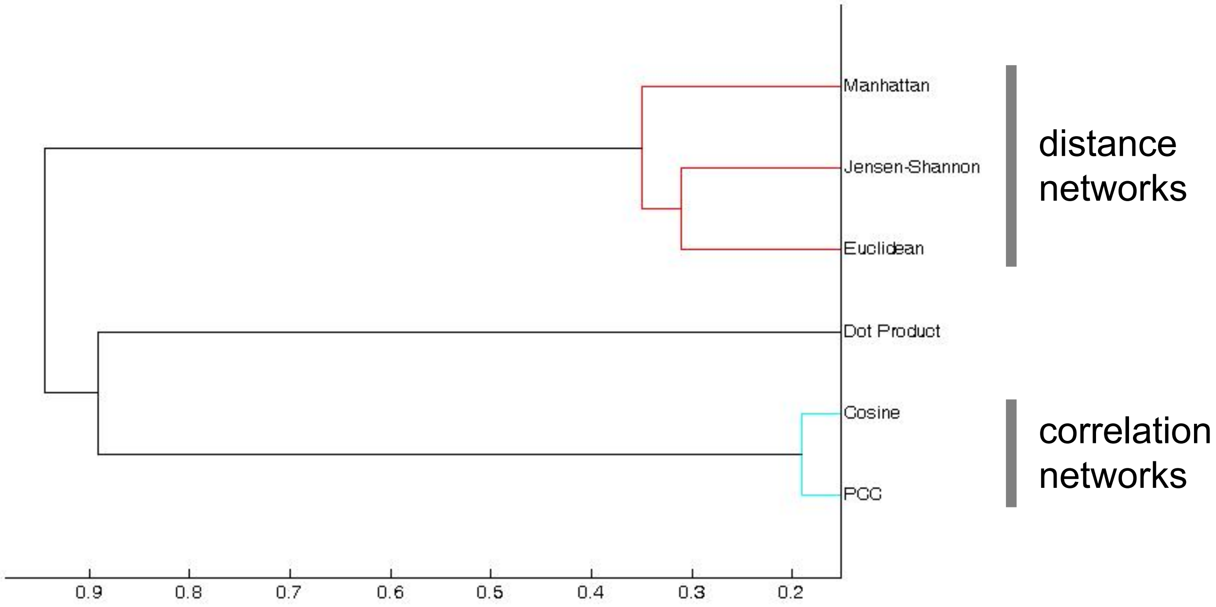

Supplementary Fig 1

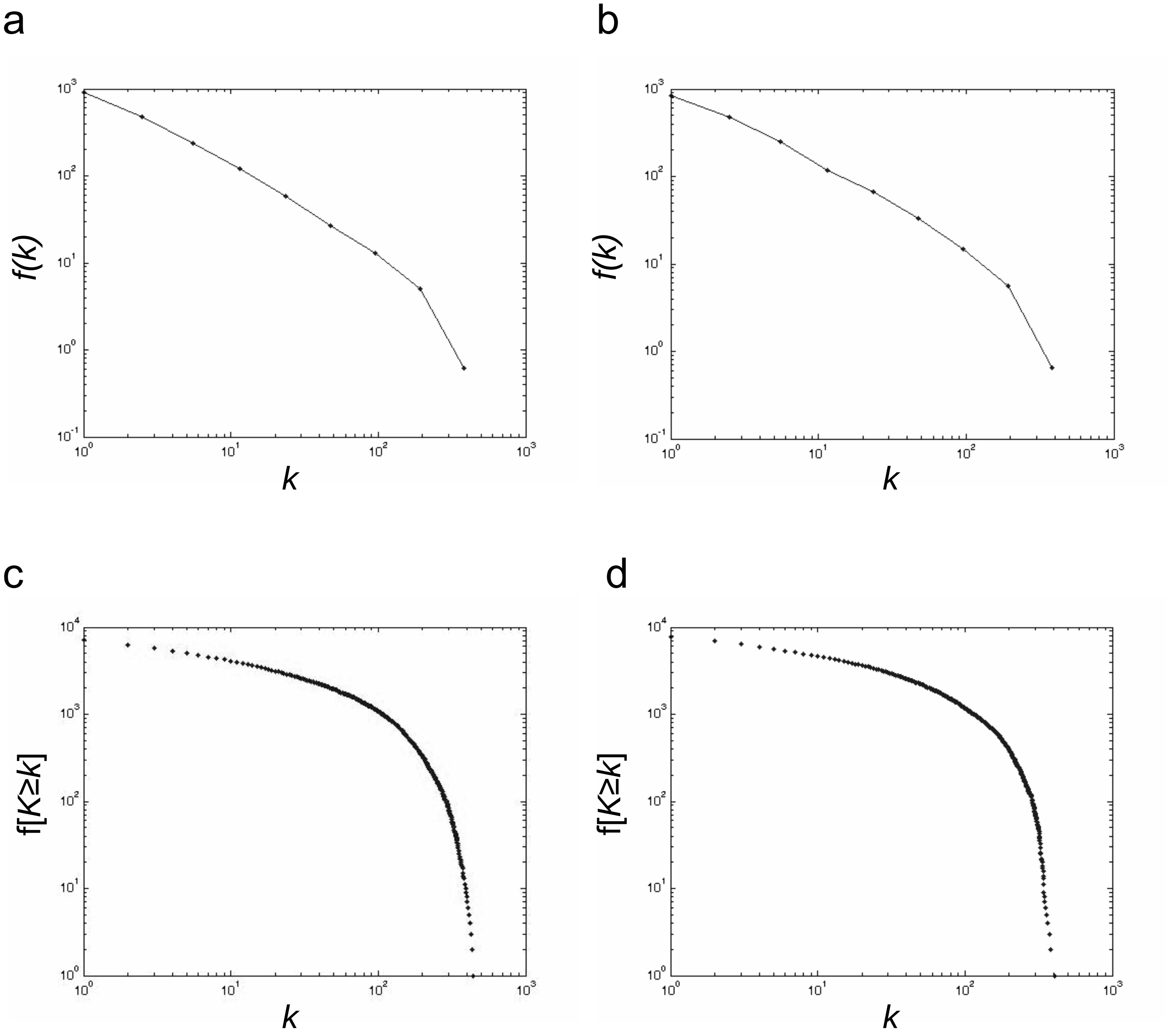

Supplementary Fig 2

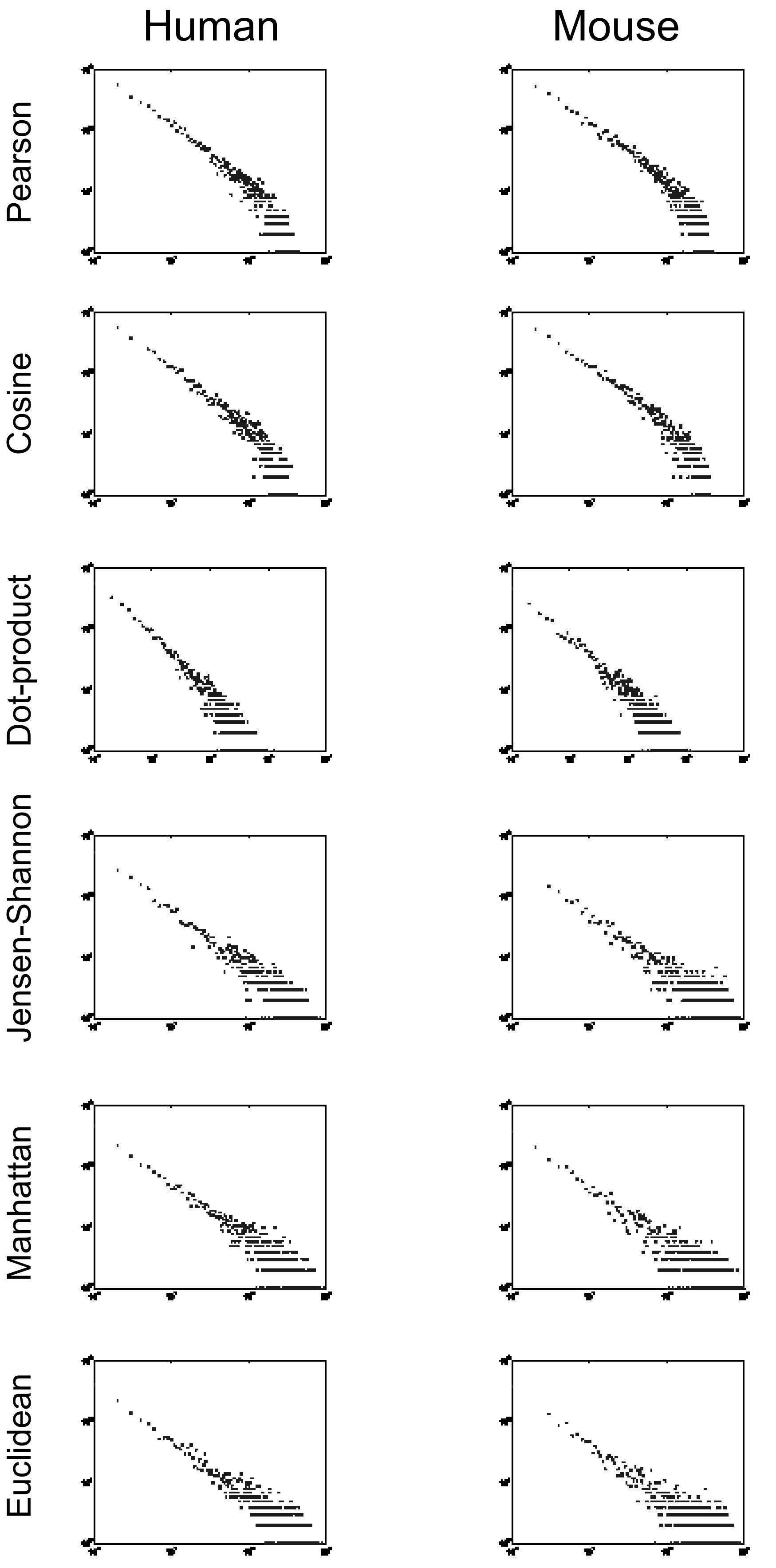

Supplementary Fig 3

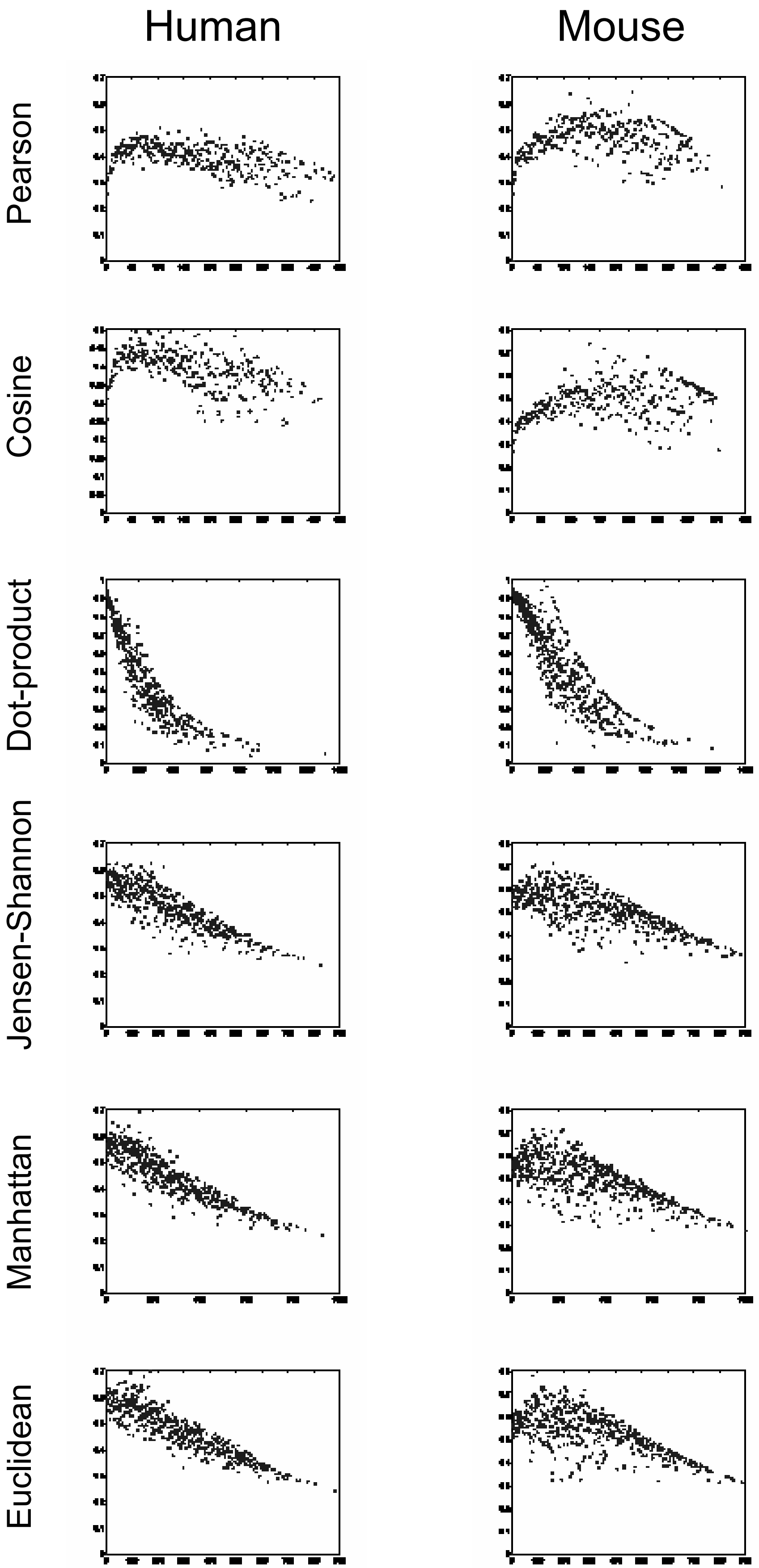

Supplementary Fig 4

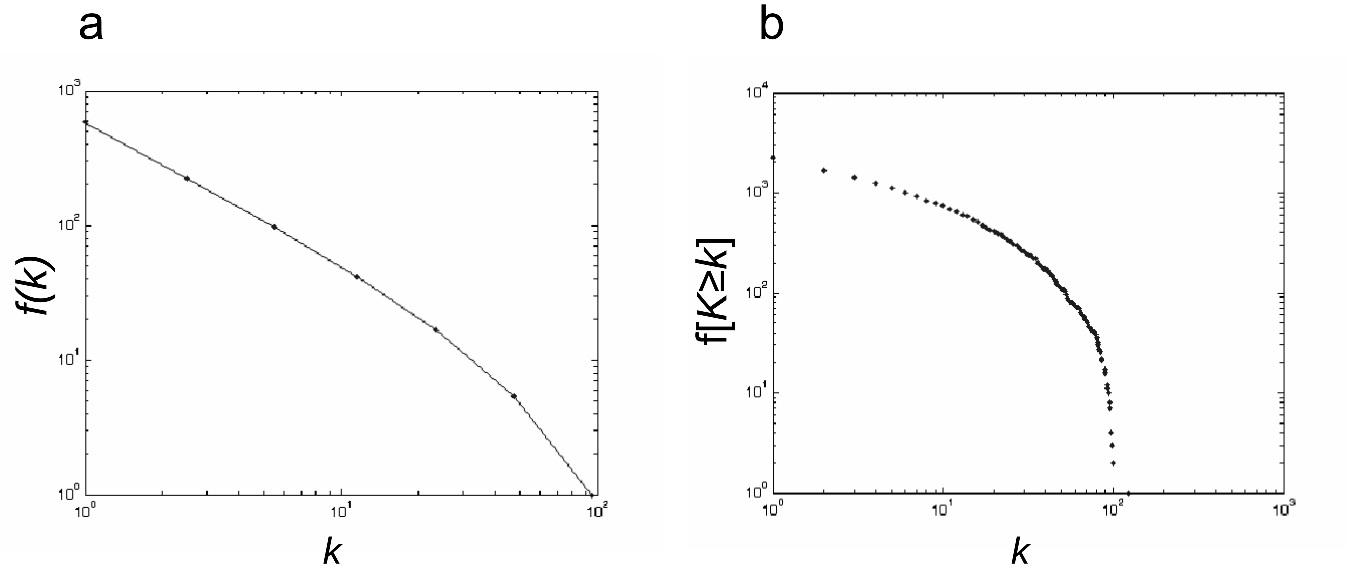

Supplementary Fig 5

a

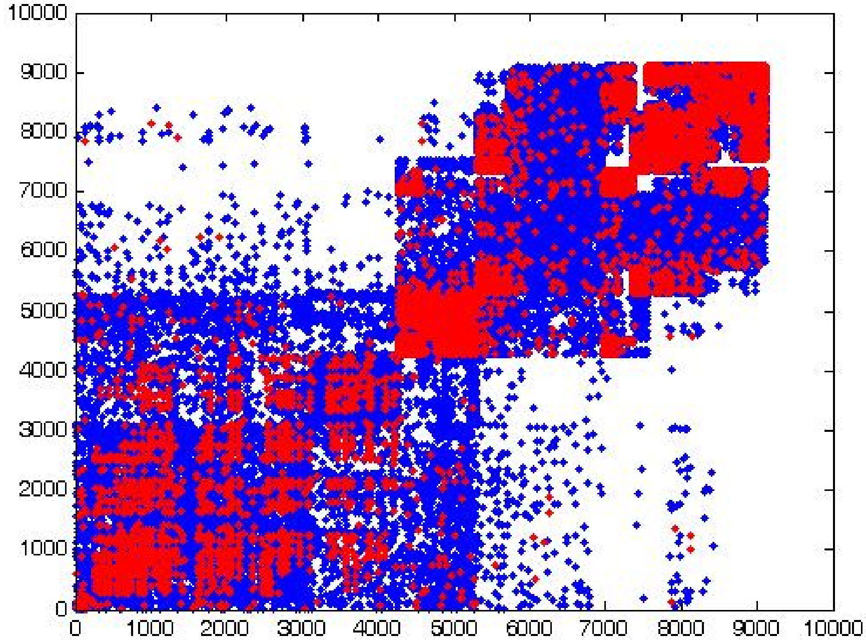

b

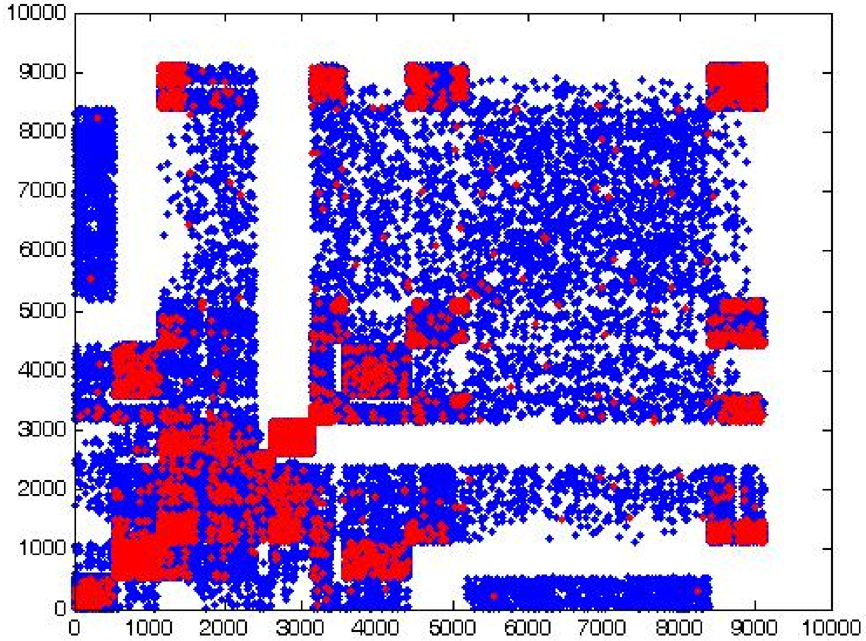

Supplementary Fig 6

a

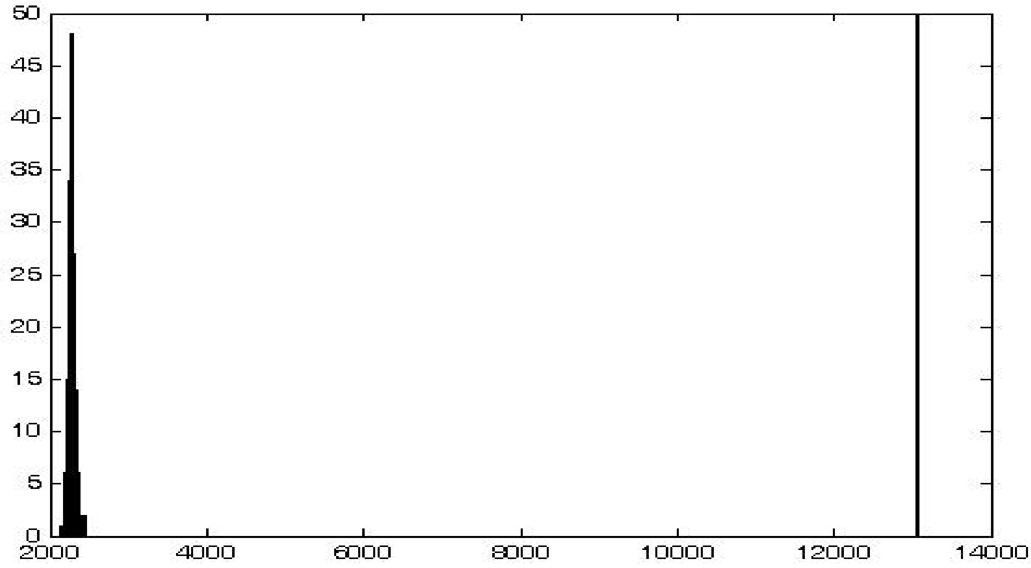

b

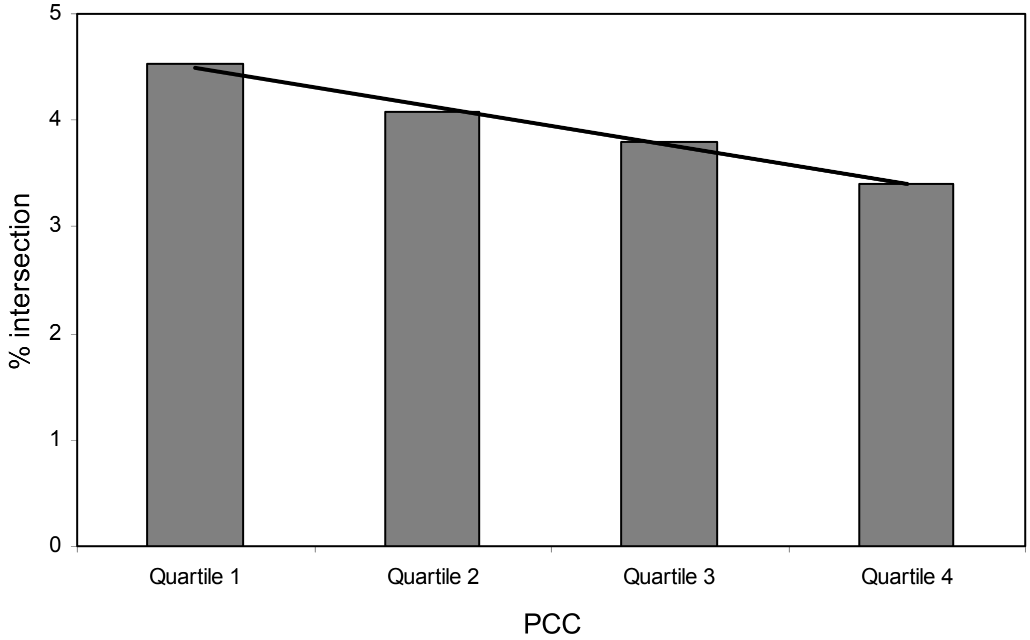

Supplementary Fig 7

a

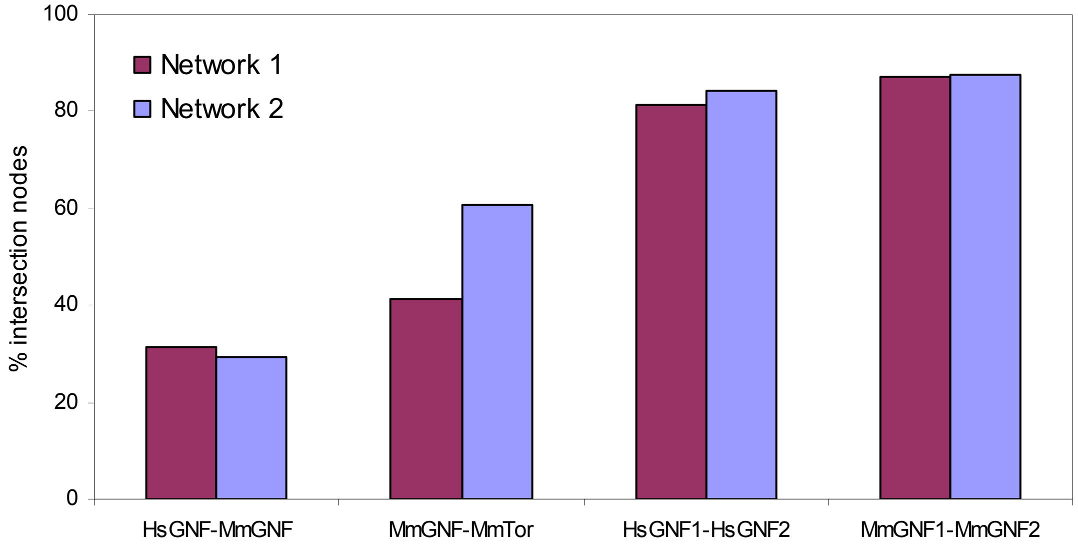

b

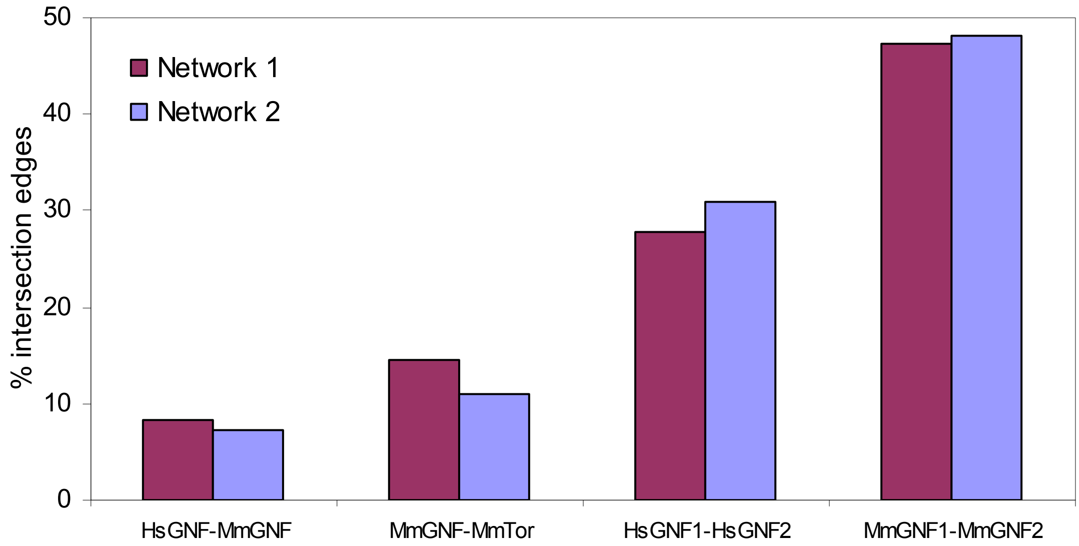

Supplementary Fig 8

a

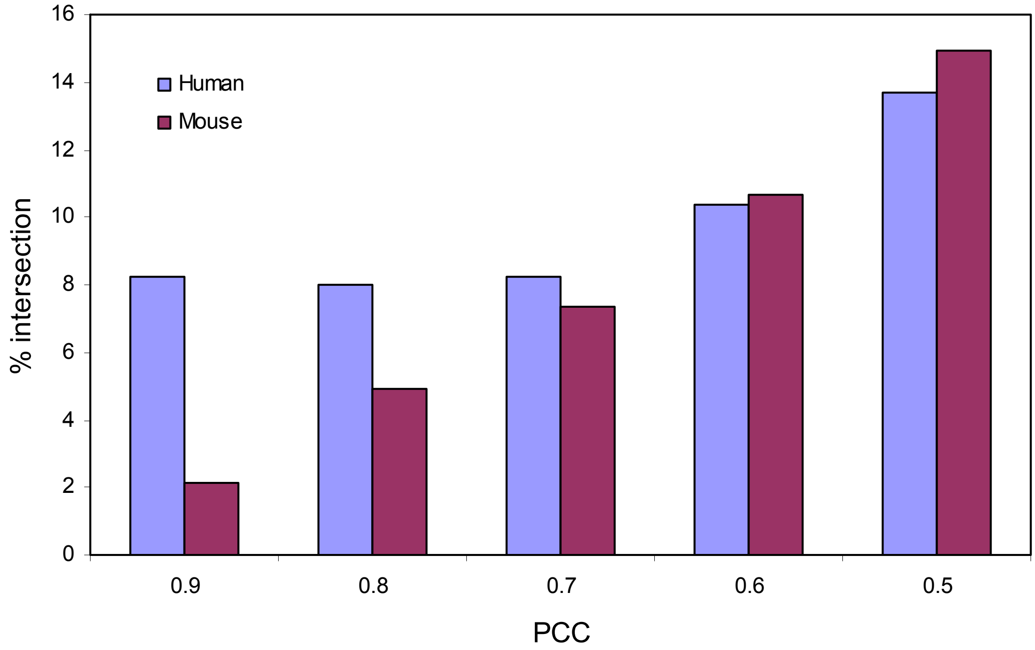

b

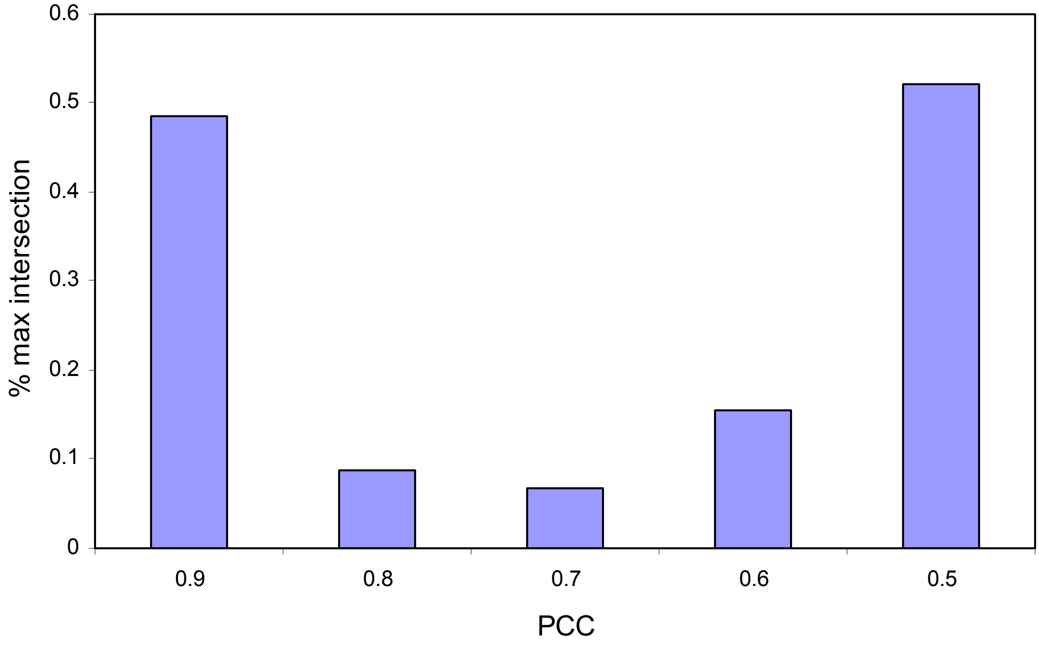

Supplementary Fig 9
